# Diagnosis of gastrointestinal parasites in reptiles: comparison of two coprological methods

**DOI:** 10.1186/s13028-014-0044-4

**Published:** 2014-08-12

**Authors:** Denis Wolf, Majda Globokar Vrhovec, Klaus Failing, Christophe Rossier, Carlos Hermosilla, Nikola Pantchev

**Affiliations:** 1 Institute of Parasitology, Justus Liebig University Giessen, Schubertstraße 81, Giessen D-35392, Germany; 2 IDEXX Vet Med Lab, Ludwigsburg D-71636, Germany; 3 Unit for Biomathematics and Data Processing, Justus Liebig University Giessen, Giessen D-35392, Germany; 4 Institute of Animal Pathologie, Vetsuisse Faculty, University of Bern, Bern CH-3012, Switzerland

**Keywords:** Reptiles, Parasites, Coproscopic diagnostic, SAF-method, Direct smear, Flotation

## Abstract

**Background:**

Exotic reptiles have become increasingly common domestic pets worldwide and are well known to be carriers of different parasites including some with zoonotic potential. The need of accurate diagnosis of gastrointestinal endoparasite infections in domestic reptiles is therefore essential, not only for the well-being of captive reptiles but also for the owners. Here, two different approaches for the detection of parasite stages in reptile faeces were compared: a combination of native and iodine stained direct smears together with a flotation technique (CNF) versus the standard SAF-method.

**Results:**

A total of 59 different reptile faeces (20 lizards, 22 snakes, 17 tortoises) were coprologically analyzed by the two methods for the presence of endoparasites. Analyzed reptile faecal samples contained a broad spectrum of parasites (total occurence 93.2%, *n* = 55) including different species of nematodes (55.9%, *n* = 33), trematodes (15.3%, *n* = 9), pentastomids (3.4%, *n* = 2) and protozoans (47.5%, *n* = 28). Associations between the performances of both methods to detect selected single parasite stages or groups of such were evaluated by Fisher's exact test and marginal homogeneity was tested by the McNemar test. In 88.1% of all examined samples (*n* = 52, 95% confidence interval [CI] = 77.1 - 95.1%) the two diagnostic methods rendered differing results, and the McNemar test for paired observations showed highly significant differences of the detection frequency (*P* < 0.0001).

**Conclusion:**

The combination of direct smears/flotation proved superior in the detection of flagellates trophozoites, coccidian oocysts and nematode eggs, especially those of oxyurids. SAF-technique was superior in detecting larval stages and trematode eggs, but this advantage failed to be statistically significant (*P* = 0.13). Therefore, CNF is the recommended method for routine faecal examination of captive reptiles while the SAF-technique is advisable as additional measure particularly for wild caught animals and individuals which are to be introduced into captive collections.

## Background

Reptiles have become increasingly common domestic pets worldwide and significant animal welfare problems are associated with pet trade [1],[2]. While several reptile species sold as pets are bred in captivity, others are taken from the wild or are the offspring of wild-caught reptiles. Particularly, exotic reptiles originating from the wild can often be infected with a variety of different invasive parasites including zoonotic ones, such as the pentastomids *Armillifer armillatus*[3],[4] and *Porocephalus* spp. [5],[6], as well as the cestodes *Spirometra* spp. [7]–[9]. Reptiles harbour a broad spectrum of internal parasites, including diverse species of protozoans, nematodes, cestodes, pentastomids, acanthocephalans and trematodes [10]–[17]. Accurate coprological examinations for reptile parasite stages are an important part of the daily routine for veterinarians to ensure the health and well-being of these animals [16],[18].

Reptile parasite detection depends on the collection of the correct specimens, the number of specimens submitted, fixation, processing methods as well as diagnostic tests to be used, and the examination of personnel who are well trained in the identification of organisms [19]–[21]. A variety of coprological methods can be applied for this purpose, including native examination, stained smears, flotation and sedimentation techniques [19],[21],[22]. Samples may be conserved with different fixatives which are mainly based on formalin as preservative agent like sodium acetate acetic acid formalin (SAF) or merthiolate-iodine-formaldehyde (MIF) [23],[24]. It should be noted, that these methods were developed for examination of humans and domestic animals (i.e. mostly mammals) and that reptile faeces show some differences compared to other domestic animals, like the quantity available for examination (generally small) or the faecal composition (presence of urates, food artifacts or soil when samples are collected from terraria).

Each one of these procedures shows its particular advantages and limitations. Direct unfixed faecal smears are used to identify motile protozoan trophozoites (flagellates, ciliates and amoebae) or other structures that float poorly (reptile specific tapeworm eggs, trematode eggs, nematode larvae) or heavy nematode eggs, as e.g. spirurids of the subfamily Physalopterinae [21],[25]. The technique is the only one which allows the evaluation of trophozoites motility (as they are readily distorted by flotation solutions due to osmotic stress), but clearly lacks good sensitivity for other parasitic stages and requires almost fresh samples [14],[19],[26]. A drop of Lugol’s iodine will enhance the internal structures of protozoan cysts (e.g. nuclei of amoebae) but will also kill present trophozoites [25],[27]. Flotation techniques allow the removal of debris and a concentration of all parasitic stages with a specific gravity lower than that of the flotation solution (nematode and cestode eggs as well as coccidian oocysts). A limitation of this technique is the missing ability to recover heavy stages like trematode eggs, large ciliate cysts and nematode larvae [22]. The SAF- and MIF-techniques allow the conservation of faecal samples for a prolonged period of time. Being sedimentation techniques, they are considered the method of choice for recovering heavy eggs (e.g. spirurid eggs as Physalopterinae or fluke eggs as e.g*. Spirorchis,Styphlodora* or *Halipegus* spp.) which do not float well because of their high specific gravity. Nevertheless, according to some authors they should also be used especially for identification of protozoan parasites [16],[28] or serve as ‘all-round-method’ for all parasite stages [24],[29]. Another recently established method (FLOTAC) has been shown to be a sensitive technique for diagnosis of parasitic infections in reptiles [30] but requires a specially developed apparatus.

In this study we compare the SAF-technique (SAF) which is used as the standard routine for reptile samples at the Institute of Parasitology in Giessen, Germany versus a combination of native smear, iodine stained smear and flotation with zinc chloride/sodium chloride solution (CNF) which is the standard approach at IDEXX Vet Med Lab for the diagnosis of gastrointestinal parasites in captive and wild-caught reptiles and highlight their respective advantages. The aim of the present study was not to compare the techniques within CNF, e.g. direct saline smear and flotation against each other in regards to their sensitivity for nematode eggs and coccidian oocysts as both techniques were used complementary. Whether similar results might be achieved for certain reptile species using less labor resources (if appropriate), by e.g. omitting completely the flotation technique, should be the goal of future studies.

## Methods

### Samples

Coprological samples from 59 different reptiles (lizards *n* = 20; snakes *n* = 22; tortoises *n* = 17) belonging to at least 27 different reptile species (in some samples, no further identification than genus level was obtainable, see Tables 1, 2 and 3) from 13 families were collected for the comparison of two different approaches for the diagnosis of intestinal parasites in reptiles. Criteria for selection were a sufficient amount of faeces for all examinations (approximately 3–4 g) and an acceptable condition (not desiccated, no gross contamination with sand/soil). In order to receive as broad an endoparasite spectrum as possible it was attempted to collect samples from a multitude of different reptile species and include captive bred animals as well as wild-caught reptiles.

**Table 1:** Reptile species investigated within group A (obtained from routine diagnostic at IDEXX Vet Med Lab) and respective results obtained by applied two methods

**Reptile number/common name**	**Reptile species (scientific name)**	**CNF**		**SAF**
		**Direct smears**	**Flotation**	
1/tortoise	Unspecified	TRC	OXY	negative
2/tortoise	Unspecified	TRC	OXY	negative
3/bearded dragon	*Pogona vitticeps*	ENTA	OXY	negative
4/hermann's tortoise	*Testudo hermanni*	TRC	OXY	OXY
5/bearded dragon	*Pogona vitticeps*	-	ISA	ISA
6/bearded dragon	*Pogona vitticeps*	-	OXY, MIL	MALO
7/tortoise	Unspecified	TRC, OXYL	OXY	OXY
8/tortoise	Unspecified	TRC	negative	ENEM
9/bearded dragon	*Pogona vitticeps*	OXYL	OXY	negative
10/tortoise	Unspecified	TRC	OXY	OXY
11/tortoise	Unspecified	TRC, BAL	negative	BAL
12/tortoise	Unspecified	TRC, BAL, ENTA	negative	negative
13/tortoise	Unspecified	TRC, NYC	ANH, OXY	NYC, ANH, OXY
14/tortoise	Unspecified	BAL	OXY	BAL
15/tortoise	Unspecified	ENM, CIL, ENEM	ENEM	ENEM
16/tortoise	Unspecified	TRC, BAL	OXY	negative
17/tortoise	Unspecified	-	OXY	OXY
18/ball python	*Python regius*	-	HET, CAP, EIMN, MYO	HET, CAP, STE, EIMN, MYO, HYN
19/tortoise	Unspecified	NYC, OXYL	OXY	BAL, OXY, OXYL
20/hermann's tortoise	*Testudo hermanni*	ENTV	OXY	ENTV, ENTA
21/tortoise	Unspecified	TRC	OXY	OXY
22/snake	Unspecified	-	EIMN	ENTM

-: no detection of flagellates, ciliates, amoebae, tapeworm/trematode eggs and nematode larvae; summary of result abbreviations in alphabetical order for Table 1, 2, 3: **ACA** (*Acanthocephala*-like eggs), **ANH** (*Angusticaecum holopterum* eggs), **ASK** (ascarid eggs), **BAL** (*Balantidium* cysts/trophozoites), **CAR** (*Caryospora* oocysts), **CAP** (capillarid eggs), **CAPN** (*Capillaria hepatica*-like eggs, rodent-specific), **CEI** (*Choleoeimeria* oocysts), **CIL** (free-living ciliates), **DTR** (digenean trematode eggs), **EIM** (*Eimeria* oocysts), **EIMN** (*Eimeria* oocysts, rodent-specific), **ENEM** (eggs/larvae of free-living nematodes), **ENM** (enteromonad trophozoites), **ENTA** (*Entamoeba* cysts, eight nuclei), **ENTV** (*Entamoeba invadens*-like cysts, four nuclei), **ENTM** (*Entamoeba muris*-like cysts, rodent specific), **HET** (heterakid eggs), **HYN** (*Hymenolepis nana* eggs, rodent specific), **HYD** (*Hymenolepis diminuta* eggs, rodent-specific), **ISA** (*Isospora amphiboluri* oocysts), **ISO** (*Isospora* oocysts), **KAPS** (*Kapsulotaenia* egg clusters), **MALO***Malamoeba cysts*, invertebrate specific, **MIL** (environmental/food/storage mites or their eggs), **MYO** (rodent specific fur mites, *Myocoptes*/*Myobia*, or their eggs), **NYC** (*Nyctotherus* cysts/trophozoites), **OXY** (oxyurid eggs), **OXYL** (oxyurid larvae), **OXYN** (rodent specific oxyurid eggs, *Aspiculuris*/*Syphacia*), **PEN** (pentastomid eggs), **SAR** (*Sarcocystis* sporocysts), **SPI** (spirurid eggs), **STE** (strongylid-type egg), **STL** (strongylid-type larvae), **STS** (*Strongyloides* eggs), **STSL** (*Strongyloides* larvae), **TRC** (trichomonad trophozoites).

**Table 2:** Reptile species investigated within group B (obtained from reptiles housed at the Rescue Reptile Centre Munich) and respective results obtained by applied two methods

**Reptile number/common name**	**Reptile species (scientific name)**	**CNF**		**SAF**
		**Direct smears**	**Flotation**	
1/green python	*Morelia viridis*	KAPS	ASK, HET, STE, CAP, EIMN, MYO	BAL, KAPS, ASK, HET, STE, CAP
2/green python	*Morelia viridis*	DTR	SAR, STE, STS, SPI, HYN, MYO	SAR, DTR, STE, STS, MYO
3/green python	*Morelia viridis*	-	STE, SPI, EIMN	STE, SPI, STL
4/green python	*Morelia viridis*	-	HET, CAP, HYD, MYO	HET, STE, CAP
5/papuan monitor	*Varanus salvadorii*	-	SPI, STS	SPI, STL
6/green python	*Morelia viridis*	-	STE	STE, STL
7/spotted tree monitor	*Varanus similis*	-	STE	STE
8/white-lipped python	*Leiopython albertisii*	-	STE, STS, EIMN, MYO	STE, STS, STL
9/timor python	*Python timoriensis*	-	STE	STE
10/emerald tree monitor	*Varanus prasinus*	-	SPI	SPI
11/green python	*Morelia viridis*	-	CAP, CAPN	CAP, STL, CAPN, MYO
12/green python	*Morelia viridis*	-	STE, EIMN	STE
13/green python	*Morelia viridis*	-	HET, CAP, STE, EIMN, MYO	HET, CAP, STE, MYO
14/green python	*Morelia viridis*	-	STE, MYO	STE, EIMN
15/green python	*Morelia viridis*	-	HET, MYO	DTR, HET, MYO
16/green python	*Morelia viridis*	DTR	HET	DTR, HET, STE
17/green python	*Morelia viridis*	-	ASK, STE, EIMN	HET, STE, STL, EIMN
18/green python	*Morelia viridis*	-	HET, SPI, CAP, MYO	SPI, ACA, MYO

-: no detection of flagellates, ciliates, amoebae, tapeworm/trematode eggs and nematode larvae; summary of result codes in alphabetical order: see Table 1.

**Table 3:** Reptile species investigated within group C (collected directly at the reptile owners domiciles in Switzerland) and respective results obtained by applied two methods

**Reptile number/common name**	**Reptilespecies (scientific name)**	**CNF**		**SAF**
		**Direct smears**	**Flotation**	
1/leaf-tailed gecko	*Uroplatus* sp.	NYC, DTR, STSL	STS, EIM, MIL	DTR, STL
2/spur-thighed tortoise	*Testudo graeca*	TRC	OXY, ENEM	OXY, ENEM
3/north african spiny-tailed lizard	*Uromastyx acanthinura*	STSL	OXY	OXY
4/plumed basilisk	*Basiliscus plumifrons*	-	OXY	DTR, OXY
5/mountain horned dragon	*Acanthosaura armata*	TRC	CAP, HET	CAP
6/ethiopian mountain adder	*Bitis parviocula*	TRC	OXY, MYO	DTR
7/black-mouthed mamba	*Dendroaspis polylepis*	-	CAR, SAR	CAR, SAR
8/kuhl's flying gecko	*Ptychozoon kuhli*	-	ISO, OXY, PEN	PEN
9/chinese water dragon	*Physignathus cocincinus*	DTR	SPI	DTR, HET
10/leopard gecko	*Eublepharis* sp.	-	OXY	negative
11/leopard gecko	*Eublepharis* sp.	DTR	negative	DTR
12/suriname redtail boa	*Boa c. constrictor* Suriname	-	MYO	STSL, MYO
13/chuckwalla	*Sauromalus obesus*	-	OXY	BAL, OXY
14/schneider's skink	*Eumeces schneideri*	-	CEI, OXY, HET	CEI
15/brook's house gecko	*Hemidactylus brookii*	-	OXY, PEN	EIM, DTR, OXY, PEN
16/jackson's chameleon	*Trioceros jacksonii*	-	CEI, HET	DTR
17/desert horned viper	*Cerastes cerastes*	-	OXYN	HYN
18/water monitor	*Varanus salvator*	-	OXYN	negative
19/malayan pit viper	*Calloselasma rhodostoma*	-	MYO	STL, MYO

-: no detection of flagellates, ciliates, amoebae, tapeworm/trematode eggs and nematode larvae; summary of result codes in alphabetical order: see Table 1.

Samples were obtained from routine diagnostic at IDEXX Vet Med Lab (*n* = 22; Group A; Table 1), from reptiles housed at the Rescue Reptile Centre Munich (*n* = 18; Group B; Table 2), and from animals taken directly at their owners' homes in Switzerland (*n* = 19; Group C; Table 3). Samples of Group A were collected during 2011 by veterinarians from different European countries (Germany, *n* = 15; Austria, *n* = 4; France, *n* = 2; Denmark, *n* = 1), and were submitted to the IDEXX Vet Med Lab for routine faecal examination for endoparasites (excluding *Cryptosporidium* spp.). Samples of Group B were obtained in 2011 from reptiles (mainly green pythons/*Morelia viridis*) which had been recently imported from Indonesia and had been taken into custody by local authorities to clarify whether they really were captive-bred individuals as declared upon import. Based on results from faecal parasitological examination and supported by further evidence obtained by physical and other laboratory examinations, strong evidence could be provided that many of these animals were indeed wild-caught [31],[32]. Samples of Group C were collected during 2011 directly at the reptile owners' domiciles in Switzerland, as part of a Master thesis at the Vetsuisse Faculty of the Universitiy Bern with the preselection to be wild-caught animals. All sample procedures were conducted in strict accordance with the German and Swiss animal protection law and by institutional review board approved protocols. All faecal samples were obtained and examined with the agreement of the owners or local authorities who where entrusted with the custodial care of the animals.

### Parasitological examination

Samples were examined immediately upon arrival at IDEXX Vet Med Lab by native and iodine-stained direct smears. Half of the remaining sample was then further processed by a faecal flotation method, while the other half was transferred to separate 12 ml sampling tubes filled with 10 ml of SAF-solution and sent to the Institute of Parasitology, Justus Liebig University Giessen, Germany.

### Combined faecals smears/flotation

Direct wet (saline) smears were prepared by mixing a small amount of faeces with a drop of 0.9% NaCl-solution on a microscope slide. Saline should be used because water can destroy protozoan trophozoites. A coverslip (22×22 mm) was placed at one end of the slide and used to push large particles of debris away and provide a uniform suspension under the coverslip. Microscopic examination was performed at 100× and 400× magnification. Iodine-stained smears were prepared likewise but a drop of Lugol's solution was added instead of saline. A conventional flotation method was performed according to Mehlhorn et al. [33]. Flotation solution was produced by mixing 800 ml distilled water, 210 g NaCl (>99.9%; Roth, Karlsruhe) and 220 g ZnCl_2_ (>97%%; Roth, Karlsruhe) and adjusting the specific gravity to 1.3 with a density hydrometer. Each sample was homogenized thoroughly on a vortexer in 50 ml preparation tubes (with sealing cap) with approx. 15 ml of the zinc chloride/sodium chloride solution. The suspension was sieved through a strainer into a second 12 ml centrifuge tube, filled almost entirely and centrifuged for 8–10 min at 300 *g*. Afterwards the tube was filled carefully with flotation solution to form a convex meniscus at the top. After 10 min a coverslip was placed cautiously in contact with the meniscus, lifted off and placed on a glass slide for microscopic examination. The cover glass was screened at 100x magnification in a meandering pattern. Suspicious structures, if necessary, were evaluated at a higher magnification.

## SAF-method

SAF-solution was prepared by mixing 15 g sodium acetate (Merck no. 1.06265. 1000), 20 ml glacial acetid acid (Merck no. 1.00056. 100), 40 ml formaldehyde (37%) and 925 ml tap-water. SAF-preserved samples were processed according to Bauer [22]. Each sample was homogenized in the SAF-filled 12 ml sampling tube used for fixation by shaking thoroughly and, if necessary, with the use of an applicator stick. Suspension was strained through gauze into a 12 ml conical tube and centrifuged for 1 min at 600 *g*. The supernatant was discarded, the sediment re-suspended in 7 ml 0.9% NaCl-solution and 3 ml of ethyl ether (Merck) and afterwards centrifuged for 3 min at 600 *g*. The plug of faecal debris on top of the saline layer was ringed with an applicator stick and the supernatant removed again. The remaining sediment was stirred up using a Pasteur pipette, then 1 or 2 drops were transferred to a slide and mounted with a coverslip (22×22 mm) for microscopic examination. Slides were completely screened at 100× magnification. If necessary, suspicious structures were evaluated at a higher magnification, and additionally a partial screening at 400× magnification for the presence of intestinal protozoa was performed.

For each method one of the authors, experienced in their respective method, examined all samples independently. The results of the SAF examination were produced after those of direct smears and flotation and were evaluated blinded, without knowledge of previous results. All parasitic stages found in any of the samples were recorded. This also included “pseudoparasites”, (or better “gastrointestinal pass through organisms”), i.e. parasitic stages from other animals than the investigated species. As a consequence of the reptile’s predatory behavior, endo- and ectoparasites from all potential prey animals can be found as transiting parasites in the intestinal tract. Accurate identification of pseudoparasites as well as free-living organisms secondarily invading the faecal sample represents a challenge to all technicians working in this field and was thus included in the spectrum of possible results. Pseudoparasites were identified by means of morphologic criteria of eggs or oocysts according to Pantchev [25].

Numbers of different parasitic stages are routinely recorded in a semi-quantitative scale, but for comparison of the two diagnostic methods the results were reduced to a qualitative statement, i.e. a positive or negative finding. For each sample, results from both methods were compared and the sample classified in one of the following categories: (i) identical outcome in both methods (ii) higher number of positive results in CNF (iii) higher number of positive results in SAF and (iv) equal number of positive results in both methods but different types of diagnosed parasites. Furthermore CNF and SAF were evaluated against each other for their capacity to detect selected single parasite stages or groups of such (Table 4). Results were classified as follows: a) no parasite stages found in either method, b) parasite stages only found in CNF, c) parasite stages only found in SAF and d) parasite stages found in both methods.

**Table 4:** Differences between CNF and SAF for individual parasitic stages

**Parasite stages**	**Number of samples**				**Statistical results**		
	**Negative in both tests**	**Only positive in CNF**	**Only positive in SAF**	**Pos in CNF and SAF**	**Fisher's exact test**	**McNemar test for marginal homogeneity**	**Differences of the probabilities of detection between CNF and SAF (%)**
Oocysts	42	10	2	5	0.009	0.04	13,6
Flagellates	44	15	0	0	Ø^3^	< 0.0001	25,4
Oxyurids	35	12	0	12	< 0.0001	0.0005	20,3
Ascarids	46	5	1	7	< 0.0001	0.22	6,8
Strongylids (eggs + larvae)	41	1	5	12	< 0.0001	0.22	6,8
Nematodes^1^	32	9	1	17	< 0.0001	0.02	13,6
Trematodes	50	0	4	5	< 0.0001	0.13	6,8
‘Heavy’ parasitic stages^2^	24	6	17	12	0.09	0.03	18,6

The association between the number of parasites found in each method was tested using Fisher's exact test and the marginal homogeneity was checked with the McNemar test.

^1^Strongylid-, ascarid-, spirurid-, strongyloid- and *Capillaria* -type eggs.

^2^Larvae, trematode eggs, amoebae, ciliates.

^3^Fischer exact test no applicable.

### Statistical analysis

The statistical comparison of the two diagnostic methods was done by means of the program BiAS [34]. In a first step comparison of the detection frequency of both methods for the observed specimens in total was done with the McNemar test. For selected single specimens the statistical association between the methods was analyzed by Fisher's exact test. For these parasite stages, further comparisons were made with the McNemar test for marginal homogeneity according to Everitt with null hypothesis stating no differences of detection frequency between the two methods. Differences of detection were regarded as significant at a level of *P* < 0.05.

## Results

**Figure 1:**
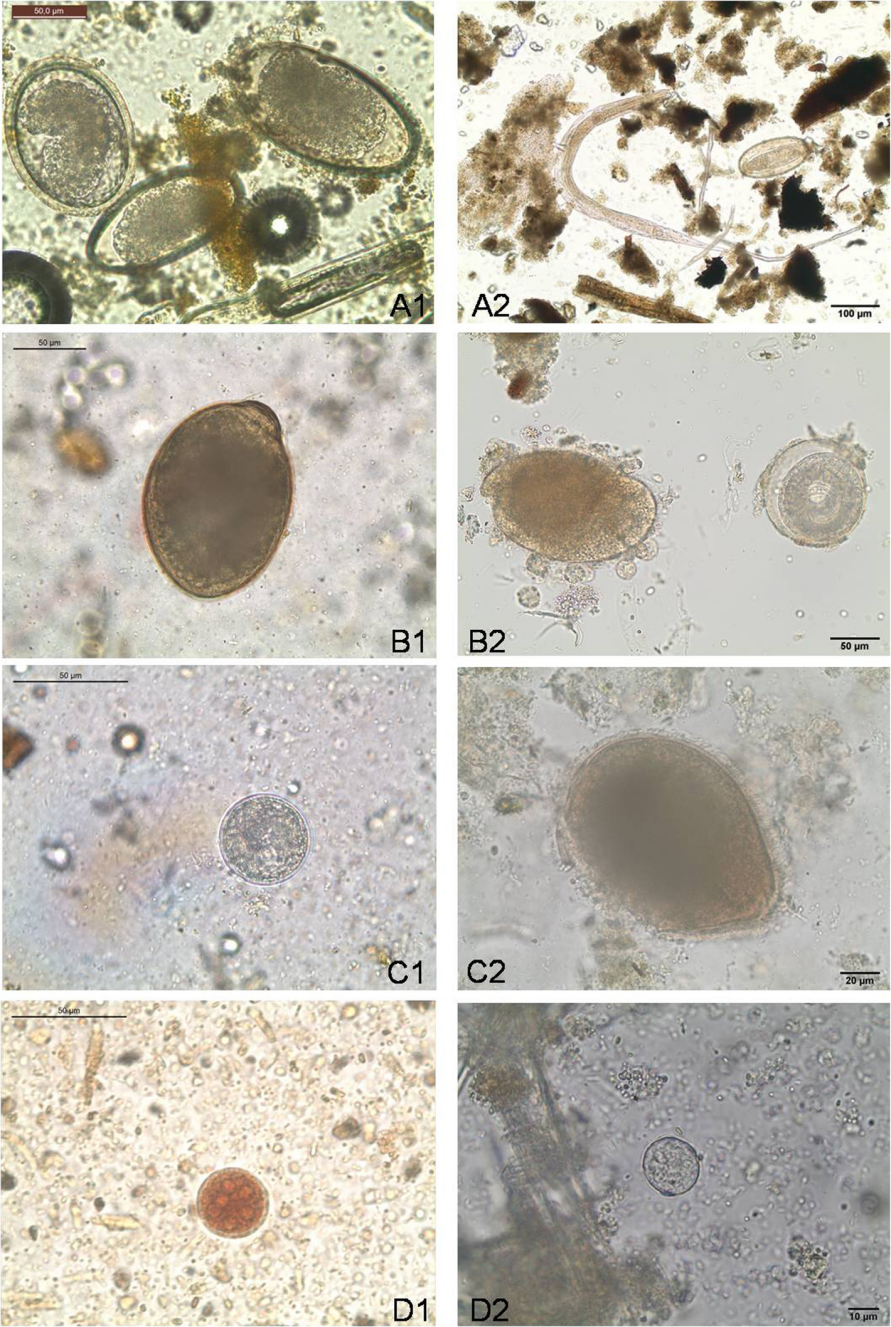
**Different parasite stages in tortoises faecal samples (with exception of D2) identified either by CNF- (left) or SAF-technique (right): A1)** ascarid egg (*Angusticaecum holopterum* (left) and two oxyurid eggs (middle, right) within a flotation **A2)** oxyurid egg and larvae **B1)** ciliate cyst (*Nyctotherus* spp.) within a direct smear, **B2)** trophozoite of *Nyctotherus* spp. (left) and embryonated ascarid egg (*Angusticaecum holopterum* right) **C1)** ciliate cyst (*Balantidium* spp.) within a direct smear, **C2)** trophozoite of *Balantidium* spp. **D1)***Entamoeba* spp. cyst (eight nuclei; not suspicious for *E. invadens*) within a iodine stained direct smear **D2)***Entamoeba* spp. cyst (eight nuclei; not suspicious for *E. invadens*) from a snake (unspecified).

**Figure 2:**
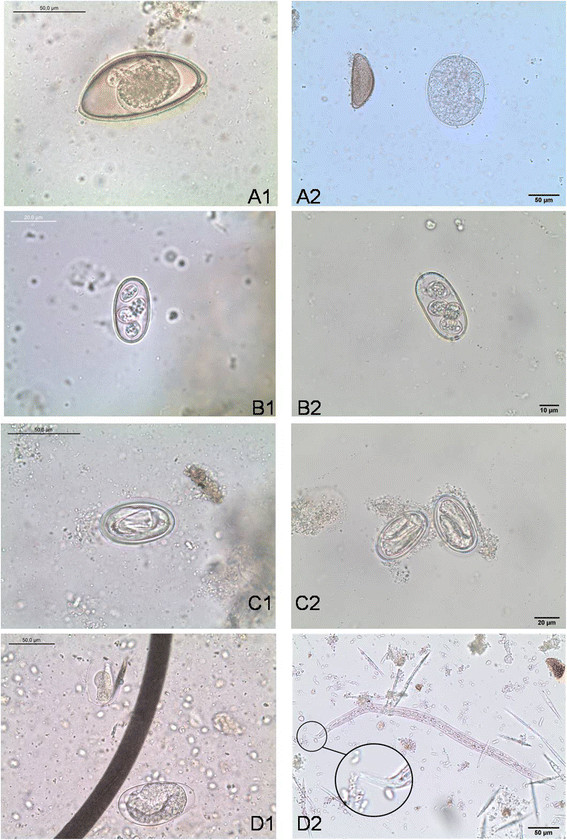
**Different parasite stages found in lizards faecal samples (with exception of D1-2) identified either by CNF- (left) or SAF-technique (right): A1)** oxyurid egg within a flotation of a Bearded Dragon (*Pogona viticeps*) **A2)** oxyurid egg (left) and pentastomid egg from a Brook’s House Gecko (*Hemidactylus brookii*) **B1)** Oocyst of *Choleoeimeria* spp. within a flotation of a Jackson's chameleon (*Triocerus jacksonii*) **B2)** Oocyst of *Choleoeimeria baltrocki* in a Schneider's Skink (*Eumeces schneideris*) **C1)** Spirurid egg (Physalopterinae) within a direct smear of an Emerald Tree Monitor (*Varanus prasinus*) **C2)** spirurid eggs (Physalopterinae) from a Emerald Tree Monitor (*Varanus prasinus*) **D1)** embryonated eggs of *Strongyloides*-type (top) and strongyle-type (*Kalicephalus*-/*Herpetostrongylus*-like; bottom) within a flotation of a Green Python (*Morelia viridis*) **D2)** infective 3rd stage larva of *Strongyloides* spp. (notice the engrailed tail-tip) from a *Boa constrictor*.

**Figure 3:**
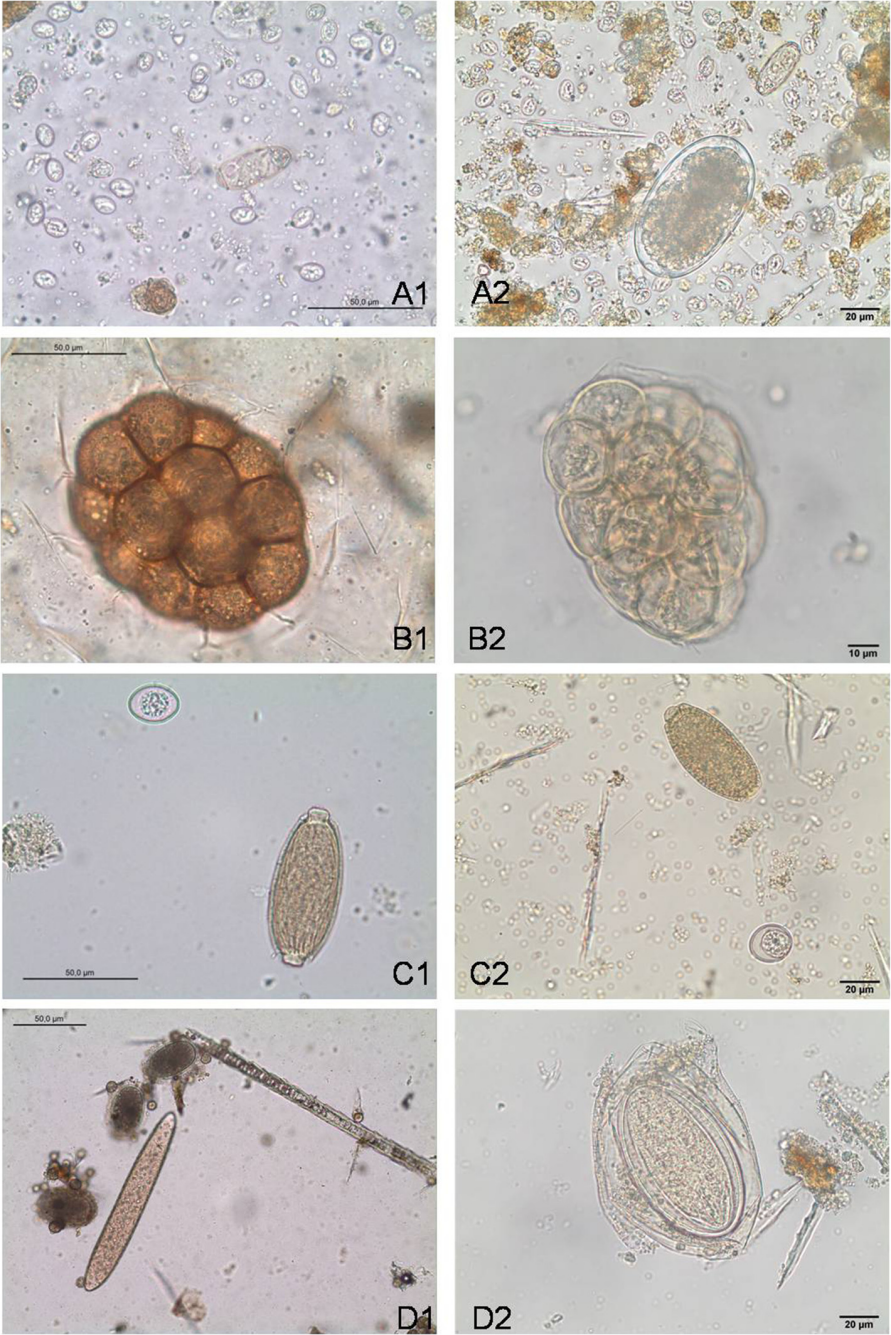
**Different parasite stages found in snakes faecal samples identified either by CNF- (left) or SAF-technique (right).***Sarcocystis* spp. sporocysts and a digenean trematode egg (middle) within a direct smear of a Green Python (*Morelia viridis*) **A2)***Sarcocystis* spp. sporocysts, a digenean trematode egg (top) and a strongyle egg (*Kalicephalus*-/*Herpetostrongylus*-like; bottom) from a Green Python (*Morelia viridis*). **B1)** Charasteristic egg cluster (*Kapsulotaenia* spp.) within a direct smear of a Green Python (*Morelia viridis*) **B2)** characteristic egg cluster (*Kapsulotaenia* spp.) in a Green Python (*Morelia viridis*) **C1)***Eimeria* spp. oocyst (rodent-specific ‘pseudoparasite’, only transiting the intestinal tract; top) and *Capillaria* (Syn. *Ophidiocapillaria*) spp. egg within a flotation of a Green Python (*Morelia viridis*) **C2)***Eimeria* spp. oocyst (rodent-specific ‘pseudoparasite’, only transiting the intestinal tract; bottom) and *Capillaria* (Syn. *Ophidiocapillaria*) spp. egg in a Royal python (*Python regius*) **D1)** Three heterakid eggs (on left) and a mite egg (*Myocoptes-musculinus*-like; middle; rodent-specific fur mite egg transiting the digestive tract) within a flotation of a Green Python (*Morelia viridis*) **D2)** unidentified egg resembling *Acanthocephala* spp. eggs in a Green Python (*Morelia viridis*).

Analyzed reptile faecal samples contained a broad spectrum of parasites including different species of nematodes (55.9%, *n* = 33), trematodes (15.3%, *n* = 9), pentastomids (3.4%, *n* = 2) and protozoans (47.5%, *n* = 28). Individual results of the parasitological examinations are shown in Tables 1, 2 and 3, and an overview of different parasite stages found in this study is shown in Figures 1, 2 and 3. Overall occurrence of ‘true’ parasitic stages (i.e. excluding pseudoparasites) was 93.2%. Pseudoparasites detected in the present study were mainly rodent-specific parasitic stages only transiting the intestinal tract of reptiles, e.g. coccidian oocysts (*Eimeria* spp., Figure 3), oxyurid eggs (*Aspiculuris*/*Syphacia)*, capillarid eggs (*Capillaria hepatica-*like), tapeworm eggs (*Hymenolepis nana*/*H. diminuta)* and fur mites or eggs (genera *Myocoptes*/*Myobia*, Figure 3*)* as well as amoebae cysts with eight nuclei (*Entamoebamuris*-like, Figure 1). Also free-living organisms (nematode stages or protozoa, e.g. ciliates) causing contamination of faeces after contact with soil/water as well as food and storage mites (and their eggs) were detected. Co-infections of two or more parasites occurred in 64.4% of the samples and wild-caught animals harboured different parasite stages than captive reptiles including two unidentified eggs from a green python (*Morelia viridis*; Figure 3).

Different results from the two diagnostic methods were obtained in 88.1% (*n* = 52, 95% confidence interval [CI] = 77.1 - 95.1%) of all examined samples, in 7 cases (11.9%) both methods rendered identical results. In 32 samples (54.2%) the combination of direct wet smears and flotation found a wider range of parasite stages than the SAF-method and conversely in 8 samples (13.6%) the SAF-technique proved superior. In 12 samples the outcome was undetermined as both methods found the same number of different parasites but without giving identical results. McNemar test for paired observations showed highly significant differences of the detection frequency between both methods as a whole (*P* < 0.0001). Furthermore, there was a significant discordance of the detectability of individual parasite stages as could be shown by McNemar test for marginal homogeneity, considering that the marginal probabilities of each type of method should be the same. Association between both tests and differences in detecting individual parasitic stages are shown in Table 4. For some parasites like pentastomida and spirurida the number of positive samples was too low to allow a statistical comparison.

For selected single specimens most distinct differences between CNF and SAF were found for protozoan stages. SAF was not able to detect any flagellates, while CNF found 15 positive samples. The majority of detected flagellates within the direct saline smear of CNF were classified based on appearance and movement patterns [25],[35] as trichomonads (Tables 1, 2 and 3). Oocysts of coccidia were present in 17 samples; 5 were detected equally by both methods, but in 10 cases oocysts were found only with CNF, and in 2 samples only with SAF (difference statistically significant with *P* = 0.04). The second highest disparity between both methods pertained the detectability of nematode eggs. This included all kinds of eggs other than oxyurids, like strongylid-, ascarid-, spirurid-, strongyloid- and Capillaria-type eggs. Though most positive samples were detected by both methods (*n* = 17), only 1 positive sample was detected by SAF alone, while vice versa 9 samples were detected only by CNF. This advantage of the CNF procedure relied mainly on a superior detection of oxyurid eggs. CNF found the double amount of positive results compared to SAF, and in no case SAF could find oxyurid eggs, as contrary to CNF. On the other hand, parasitic stages with a high specific gravity were detected significantly more often by SAF (*P* = 0.03). This also accounted for trematode eggs, but this finding did not prove statistically significant (*P* = 0.13).

## Discussion

Parasites in reptiles kept in captivity, such as zoos, farms or as domestic pets, are amongst the most frequent pathogens to be found and may induce detrimental effects on the well-being of these animals [16],[18]. Therefore appropriate diagnosis is an important issue. A variety of procedures for the diagnosis of parasite stages in coprological samples have been developed, some showing a broad range of application while others are rather specialized methods used only for certain types of parasites. Though the SAF-technique has been proposed by some authors as an “all-round” technique, this concerns mainly the examination of human samples [24],[29]. In the diagnosis of human parasites the SAF-method has been successfully applied for decades and has been subject to numerous evaluations, but so far no comparison with other procedures for the examination of reptile faeces has been done. A way to overcome the limitations of a single method is to apply several techniques on a single sample, a frequent combination being a flotation method with direct faecal smear. When performed by experienced technicians, both methods will require a similar amount of labour though this may vary between individual samples. In this study we evaluated the SAF-technique versus a combination of a direct saline smear, iodine stained fresh faecal smear and flotation with zinc chloride/sodium chloride solution.

Both methods showed a high degree of discrepancy in the positivity rates with the combination of direct smears and flotation finding a significantly higher number of parasite stages. The first reason for this difference was based on the superior ability of CNF to detect protozoan stages, mainly coccidian oocysts (Figure 2) and flagellate trophozoites, mainly trichomonads (Table 4). While oocysts can be concentrated easily by flotation, the main advantage of a direct saline smear is the fact that active protozoans can be readily recognized by observing amoeboid, ciliate and flagellate motility [14],[19],[25]. One limitation is the fact that very little faecal material is used, and no concentration is performed. Also, as movement is the principal characteristic that allows recognition of trophozoites in this procedure, if the faecal layer is too thick, it will be hard to see small, colorless protozoa moving in the field.

In the present study no flagellates were found by means of the SAF-technique, even though a small number of samples which had been found positive in direct smears were re-examined to rule out false negative results due to subjective interpretation. The reasons for this finding remain unclear. While some authors specifically recommend the SAF-technique for detection of protozoa in reptiles [14],[16], others refer to the impairment being due to this method [23],[36]. When establishing the MIF-technique (as a predecessor to the SAF-method), Sapero and Lawless [23] stated that large numbers of flagellate trophozoites failed to become fixed or were unidentifiable. Pietrzak-Johnston et al. [36] describe that in samples preserved in SAF amoebae were difficult to identify because of poor preservation. On smears prepared from these samples and stained with iron haematoxylin organisms could not be easily recognized. On the other hand some authors found faecal preservation and subsequent staining superior to wet mount examination for detection of the trophozoite stages [28],[29]. In our own laboratory working experience at the Institute of Parasitology (JLU Giessen, Germany) in coprological samples from mice which regularly harbour flagellates, these parasites can be easily identified. Most common in mice are trichomonads, like *Tritrichomonas muris*. It is possible that these species, though equipped with delicate flagellae and an undulating membrane show higher tenacity and better fixation properties than the species found in the digestive tract of reptiles.

For the identification of protozoan trophozoites faeces should be examined as fast as possible or should be fixated immediately after sampling [19],[24],[37]. It was not possible to fully comply with this demand as samples had to be sent to the laboratory before they could be further processed and preserved. Though flagellates could still be detected in various cases in direct wet mount, the time between sampling and fixation in SAF might have sustained some precursory damage to the specimens. Furthermore, samples in this study had been stored in SAF for more than a year prior to microscopic examination and maybe this period of time proved to be too long. On the other hand, SAF solution, unlike MIF fixative, seems indefinitely stable and is thus well suited for use in collecting samples that are to be stored for long periods [24]. Test specimens prepared in SAF appear to be stable over a long time as well. No loss or deterioration of organisms was reported after six months [23],[38]. In a comparison among European reference laboratories, microscopic diagnosis of SAF-fixed stool samples for helminths and intestinal protozoans was carried out up to 12 months after sampling [39]. The authors were surprised to find a large degree of variance in the positivity rates reported by the different laboratories analyzing the same SAF-preserved stool specimens but did not see any restriction caused by the time between sampling and examination. In a second ring test there was again only moderate agreement between the centres for pathogenic intestinal protozoans while common helminths were reliably diagnosed by the participating laboratories. In the latter case the authors did raise the question whether storing time might have had an effect on the outcome of the study, but state that samples preserved in SAF for teaching purposes remained intact even for many years [40].

Other negative influences might be attributed to the fact that enzymatic activity is still present in SAF-preserved specimens [41]. Also, by some authors, the use of ether in the processing of samples is viewed critically. Faust et al. [27] found ether to shrink protozoan cysts, so that they were not suitable for proper diagnosis. Glinz et al. [42] found that prevalence of hookworm eggs decreased after stool samples had been preserved in SAF for 83 days. Also, considerably higher hookworm egg counts were revealed in SAF-preserved stool samples with no ether, whereas destroyed hookworm eggs could be observed after exposure of the sample to ether. In our own study it could be observed that some strongylid-type eggs or amoebae cysts showed an impaired shell together with a general poor appearance. In contrast, ascarid eggs can remain viable in 10% formalin for up to 8 months [43]. It is therefore common to find embryonated eggs when examining long-preserved SAF samples (Figure 1). The aspect of negative influence on delicate amoebae by fixatives and other chemicals like ether certainly remains to be further investigated.

The second reason for the better total performance of CNF was the higher sensitivity to detect nematode eggs, i.e. mainly oxyurid eggs (Table 4; Figures 1 and 2). Apparently, the flotation procedure has a higher ability to concentrate these types of eggs than the sedimentation technique. A higher sensitivity of the SAF-method would probably be achievable if the entire sediment was examined as recommended by some authors [40], but this would not have been possible without a disproportional time effort. Unless storage of the remaining sediment is required (e.g. for re-evaluation), an additional flotation step of the residual sediment could probably be useful in overcoming this limitation.

On the other hand, concerning the identification of strongylid nematodes in the present study, SAF detected a higher number of samples as positive (relying mainly on better detection of strongylid larvae), but this proved not to be statistically significant. SAF also showed advantages in the detection of the operculated eggs of digenean trematodes although the difference was not statistically significant (*p* = 0.13). All samples found positive by CNF were only detected by means of the direct smears as part of this combined method. This is not surprising, as trematode eggs show a high specific gravity [22],[44]. Generally, when counting ‘heavy’ parasitic stages (i.e. with a high specific gravity like larvae, trematode eggs, amoebae, ciliates) as one group, SAF did show significant advantages over CNF. This could be expected, as these stages will not be concentrated by flotation, so their detection is restricted to the examination of direct smears where they can be missed more easily based on the missing concentration.

Some nematode infections as oxyurids, strongylids, *Strongyloides*/*Capillaria* spp. and to some extent heterakids or ascarids (in tortoises and chameleons) show high prevalences in captive reptiles [20] and may pose a serious health problem for these animals while digenean trematodes will require one or more intermediate hosts, mainly molluscs. In terraria this cycle will most often be interrupted so that captive animals normally are not exposed to re-infection [20],[25],[31],[32]. Thus it seems advisable to examine captive reptiles by means of CNF rather than the SAF-method. Nevertheless, for examination of wild animals and individuals which are to be introduced into captive collections an additional sedimentation based method like the SAF-technique seems appropriate.

Furthermore, the detection of parasites with complicated life cycles as part of forensic parasitology gives helpful evidence for government officials dealing with legal regulations of exotic animals [45]. Large numbers of reptiles are being imported to Europe every year, and often government officials are challenged by the question whether these animals were farmed as documented in the import licence papers or taken directly from the wild [31],[32]. One such wild-caught green python (*Morelia viridis*) involved in this study harboured *Kapsulotaenia* spp. The characteristic egg packets of these cestodes could be diagnosed by both methods, but showed a rather poor and altered appearance in the SAF-fixated samples making identification more difficult (see Figure 3). The reasons might be the same as discussed in connection with diagnosis of protozoa but it can also be stated that the SAF-method generally demands higher diagnostic experience on the investigator.

In the faeces of another wild caught green python two eggs were found by means of the SAF-method which could not be identified accurately. Size and shape resembled somehow that of *Acanthocephala* species (Figure 1) found in a monitor lizard (*Varanus* sp.) [18]. Only few reptiles are known to harbour thorny-headed worms [25] and no member of this order has been described in green pythons so far. The animal sampled in this study was wild caught and imported to Germany from Indonesia. From the Indo-Australian region members of the genus *Sphaerechinorhynchus* have been reported in Black Snakes (genus *Pseudechis*, Australia), from Asian cobra, *Naja naja* (North Borneo) and from king cobra (*Ophiophagus hannah*, Southeast Asia, undocumented area) [46],[47]. Nevertheless, it cannot be ruled out that these eggs, even if truly belonging to thorny-headed worms, are not specific parasites of green pythons. Acanthocephalans are frequent parasites of birds, giving the chance that these eggs merely represent pseudoparasites, a fact that technicians examining reptile faeces should always be aware of.

## Conclusion

The SAF method showed significant differences when compared to a combination of direct saline smear, iodine stained smear and flotation with zinc chloride/sodium chloride solution (CNF) for the diagnosis of intestinal parasites of reptiles. Advantages of CNF were mainly related to the higher detectability of protozoan stages and nematode eggs while digenean trematode eggs were better diagnosed by the SAF-method. Thus, CNF is the recommended method for routine faecal examination of captive reptiles while the SAF-technique is advisable as additional measure for wild caught animals and individuals which are to be introduced into captive collections. Also, SAF remains the method of choice when samples cannot be examined immediately or re-evaluation of samples and thus fixation of faeces is required. An additional flotation step of the residual sediment could probably be a useful measure to enhance the susceptibility of this method. Also samples should still be processed as soon as possible, as impaired recovery of parasite stages cannot be ruled out after a prolonged period of time.

## Competing interests

The authors declare that they have no competing interests.

## Authors’ contributions

DW and NP have been involved in the initial design of the study, carried out parasitological examinations and wrote main parts of the manuscript. MGV and CR participated in coordination of the study, the collection of samples and data and supported parasitological examinations; KF has been the main responsible for data analysis in corporation with DW. CH supported writing and design of the study, and contributed with critical and final review of the manuscript. All authors have read and approved the final manuscript.
